# Panhypopituitarism and Central Hypothyroidism Presenting as Myoclonus and Hyperreflexia

**DOI:** 10.7759/cureus.39140

**Published:** 2023-05-17

**Authors:** Ifrah Nadeem, Wahab J Khan, Anum Nadeem, Abdul Wadood, Sudan Thapa

**Affiliations:** 1 Internal Medicine, University of South Dakota Sanford School of Medicine, Sioux Falls, USA; 2 Physiology, Rashid Latif Medical College, Lahore, PAK; 3 Internal Medicine, National Hospital and Medical Center, Lahore, PAK; 4 Endocrinology, Diabetes and Metabolism, University of South Dakota Sanford School of Medicine, Sioux Falls, USA

**Keywords:** thyrotropin, hyperreflexia, pituitary mass, central hypothyroidism, myoclonus

## Abstract

Panhypopituitarism may present with symptoms of predominantly one or more hormonal deficiencies. Central hypothyroidism usually presents with typical symptoms of hypothyroidism, such as fatigue, weight gain, menstrual abnormalities, bradycardia, thick, coarse skin, muscle fasciculations, and hyporeflexia, among others. Herein we present a case of central hypothyroidism along with panhypopituitarism presenting with unusual symptoms of tongue fasciculation, hyperreflexia, and myoclonic jerks.

## Introduction

Panhypopituitarism represents a deficiency of all pituitary hormones resulting from either pituitary gland dysfunction or hypothalamic disease. Depending on the underlying etiology, type, and severity of hormonal insufficiency, hypopituitarism may present with symptoms of predominantly one or more hormonal deficiencies. In addition, there may be symptoms from the mass effect on the brain tissues. The endocrine and nervous systems maintain homeostasis through the regulated secretion of hormones, neurotransmitters, and neuromodulators. Thyrotropin-releasing hormone (TRH) is one of those neuromodulators secreted from the hypothalamus and regulates thyroid secretion via thyroid-stimulating hormone (TSH) from the pituitary. It has been described to cause neuromuscular hyperexcitability in both humans and animals via its modifying role in synaptic functions in addition to its effect on the pituitary [1,2]. We present a case of a female patient who presented with generalized weakness, atypical neurological signs of tongue fasciculation, hyperreflexia, and myoclonic jerks more prominent in the lower extremities, later diagnosed with panhypopituitarism, central hypothyroidism, and found to have pituitary macroadenoma.

## Case presentation

A 78-year-old Caucasian woman with a past medical history of essential hypertension, left total hip arthroplasty, depression, and hyperlipidemia presented to the emergency department with generalized weakness, twitching movements of her tongue, and involuntary jerking movements of her lower limbs for one month. She also reported intermittent diffuse headaches, loss of appetite, and an unintentional weight gain of about 10-15 pounds in the last six months. The patient denied any sudden onset severe headache, numbness or tingling, vision changes, or recent memory loss. In addition, she denied any speech or gait abnormality. On admission, vital signs included a temperature of 96.9 °F, blood pressure of 115/83 mmHg, a pulse of 55 beats/min, and a respiratory rate of 18. Her BMI was 28. Neurological examination revealed tongue fasciculation and bilateral lower extremity myoclonus. In addition, her visual field testing was normal. Basic labs, including complete blood count and metabolic panel, showed no remarkable abnormalities. A chest X-ray did not show any acute abnormality. A CT scan of the head showed an apparent seller mass but ruled out any acute pathology. Her seller mass alone could not explain the neurological symptoms. Further neurologic, infectious, and rheumatologic work-up revealed negative results for antinuclear antibodies, rheumatoid factor, erythrocyte sedimentation rate, Lyme disease, and West Nile serology. In addition, an MRI of the cervical, thoracic, and lumbar spine was negative for any mass effect.

**Figure 1:**
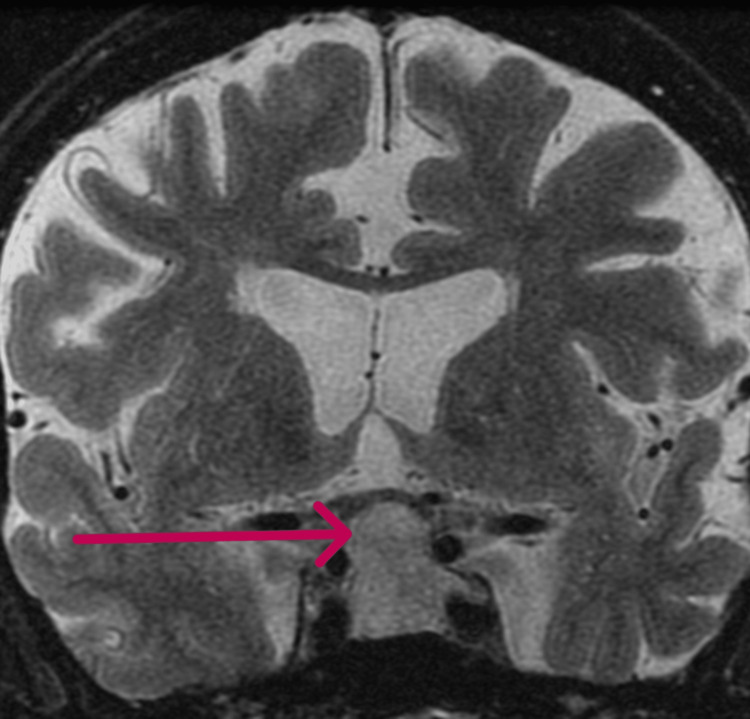
MRI image of the pituitary gland The arrow shows a large pituitary tumor pushing on the optic chiasma.

**Figure 2:**
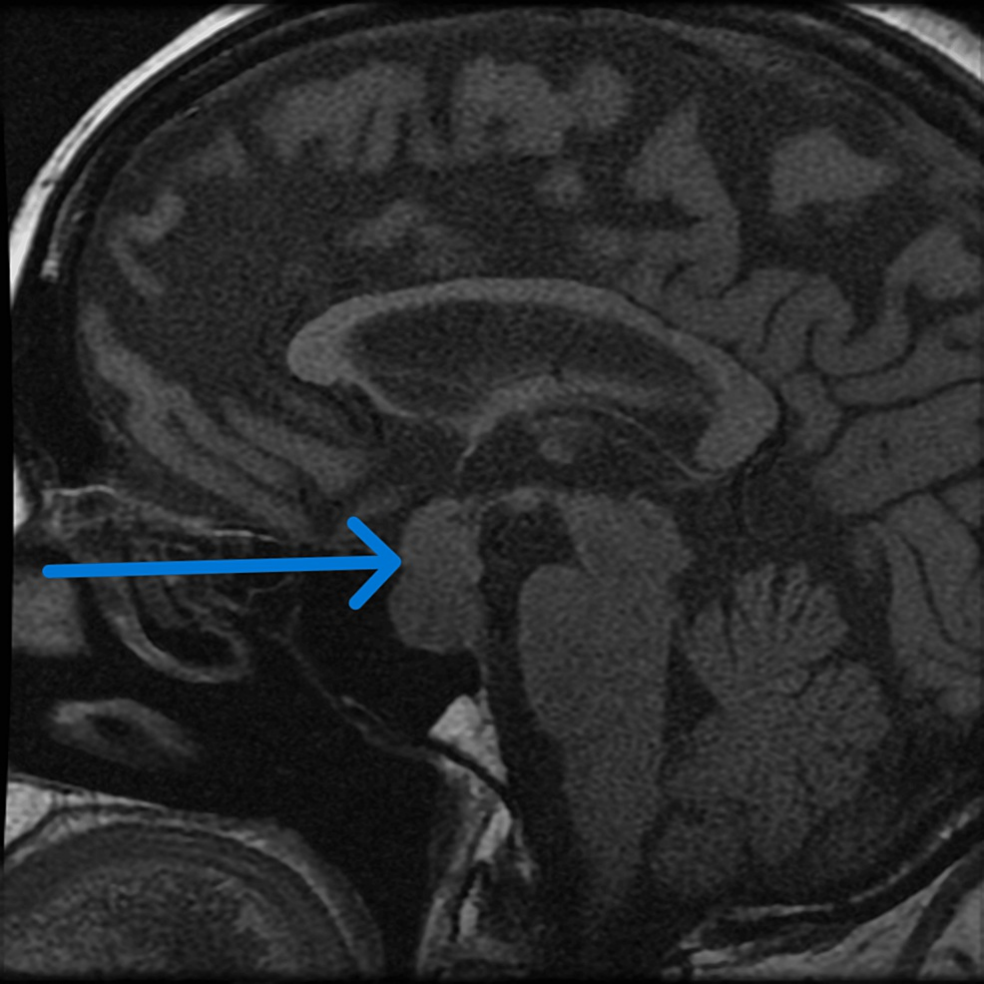
MRI of the pituitary gland in the coronal section The arrow shows the large pituitary tumor.

Subsequent Pituitary MRI with and without contrast showed a 2.0 x 1.5 x 2.3 cm sellar/suprasellar mass. The mass was mildly deforming the optic chiasm, invading the left cavernous sinus, and deviating the infundibular stalk to the right side (Figures 1, 2).

Laboratory work-up revealed secondary adrenal insufficiency, central hypothyroidism, hypogonadotropic hypogonadism, and hyperprolactinemia. Values are shown in Table 1. The patient was diagnosed with panhypopituitarism due to likely non-functioning pituitary macro adenoma. She was started on hormone replacement with levothyroxine 50 mcg daily and a stress dose of hydrocortisone, gradually tapered to 15 mg in the morning and 10 mg in the afternoon. She was also given a trial of cabergoline 0.5 mg 2 times a week for hyperprolactinemia. The patient reported significant improvement in her energy levels and colonic spasms after initiating the hormone replacement. Her repeat lab work at the outpatient endocrinology clinic showed an undetectably low prolactin level of less than 0.8 ng/ml, low TSH with normal free T4 consistent with central hypothyroidism normalized with thyroid hormone replacement. Repeat MRI after three months showed a stable 1.5 x 2.0 x 2.3 cm sellar/suprasellar mass, mildly deforming the optic chiasm and deviating the infundibular stalk. She is still undergoing evaluation for surgical resection in the near future.

**Table 1:** Endocrine laboratory values at the time of admission Abbreviations; Adrenocorticotrophic hormone (ACTH); Thyroid-stimulating hormone (TSH); Luteinizing hormone (LH); Follicle-stimulating hormone (FSH); Cortisol level test (AM Cortisol)

Lab	Initial value	Reference value
ACTH	10 pg /mL	7.2 – 63 pg/mL
AM Cortisol	1.7 ug/dL	3.7 - 19.4 ug/dL
Cortisol 1-hour post-stimulation	8.7 ug/dL	>16 ug/dL
TSH	1.1 uIU/mL	0.35-4.94 uIU/mL
Free T4	0.5 ng/dL	0.7-1.5 ng/dL
Prolactin	131.4 ng/mL	3.5-20.0 ng/mL
FSH	2.8 uIU/mL	26.7 – 133 uIU/mL
LH	<0.1 uIU/mL	5.2 – 62 uIU/mL
Estradiol	<24 pg/mL	10-28 pg/mL

## Discussion

Hypothyroidism is the functional deficiency of thyroid hormone in the body. It could be primary, secondary, or tertiary hypothyroidism. Clinical manifestations related to hypothyroidism are usually the same regardless of the etiology. However, there may be additional findings related to hypothalamic or pituitary dysfunction in the case of secondary or tertiary hypothyroidism due to non-thyroidal illness. The usual muscular symptoms related to hypothyroidism are proximal myopathic weakness, fatiguability, myalgias, pseudohypertrophy, rhabdomyolysis, and myoedema, along with delayed relaxation of muscle fibers. In addition, muscle fasciculations are common with hypothyroidism. But myoclonus and hyperreflexia are the findings in hyperthyroidism except for one hypothyroid state, i.e., Hashimoto encephalopathy (HE) [3,4]. What causes hyperreflexia and myoclonus in HE remains unclear. It is thought to be related to encephalitis present in HE, which itself is believed to be an immune-mediated disorder rather than representing the direct effect of an altered thyroid state on the central nervous system.

There have been reports of TRH playing an important role in the pathophysiology of myoclonus and hyperreflexia in patients with HE [5] and without HE [6]. Our patient presented with myoclonus and hyperreflexia. She was found to have pituitary macroadenoma causing panhypopituitarism. In addition, she had central hypothyroidism with likely elevated TRH. It is well known that TRH stimulates prolactin secretion, and the effect is exaggerated if concurrent hypothyroidism is present [7]. Our patient also had hyperprolactinemia. These findings suggest that she had central hypothyroidism along with stalk effect. TRH can play a stimulatory or inhibitory role in the brain depending on its location and the physiologic state of the body. It is analeptic in sedated subjects and anti-epileptic in conditions predisposing to seizures [8]. Therefore we suggest a high level of TRH expected in central hypothyroidism might have been the reason for her hyperreflexia and myoclonus. It is also supported by the fact that exogenous glucocorticoids and thyroid replacement decrease TRH levels. Our patient’s myoclonus and hyporeflexia improved significantly with thyroid and glucocorticoid replacement. However, more studies are required to confirm the causal relationship between TRH and neuromuscular hyperexcitability and the effects of TRH on the central nervous system.

## Conclusions

The TRH plays an important role in both endocrine pathophysiology and neurophysiology. TRH may have stimulatory or inhibitory effects in the brain depending on its location and the physiologic state of the body. It can cause neuromuscular hyperexcitability via its function as synaptic modulation in addition to its effects on the pituitary. Hypothyroidism can present with hyperreflexia and myoclonus, mostly in two low thyroid conditions, i.e., Hashimoto encephalitis and central hypothyroidism.
